# Rho inhibition by lovastatin affects apoptosis and DSB repair of primary human lung cells *in vitro* and lung tissue *in vivo* following fractionated irradiation

**DOI:** 10.1038/cddis.2017.372

**Published:** 2017-08-10

**Authors:** Verena Ziegler, Christian Henninger, Ioannis Simiantonakis, Marcel Buchholzer, Mohammad Reza Ahmadian, Wilfried Budach, Gerhard Fritz

**Affiliations:** 1 Institute of Toxicology, Medical Faculty, Heinrich Heine University Duesseldorf, Moorenstrasse 5, Duesseldorf, Germany; 2 Department of Radiotherapy and Radiation Oncology, University Hospital Duesseldorf, Moorenstraße 5, Duesseldorf, Germany; 3 Institute of Biochemistry and Molecular Biology II, Medical Faculty, Heinrich Heine University, Duesseldorf, Germany

## Abstract

Thoracic radiotherapy causes damage of normal lung tissue, which limits the cumulative radiation dose and, hence, confines the anticancer efficacy of radiotherapy and impacts the quality of life of tumor patients. Ras-homologous (Rho) small GTPases regulate multiple stress responses and cell death. Therefore, we investigated whether pharmacological targeting of Rho signaling by the HMG-CoA-reductase inhibitor lovastatin influences ionizing radiation (IR)-induced toxicity in primary human lung fibroblasts, lung epithelial and lung microvascular endothelial cells *in vitro* and subchronic mouse lung tissue damage following hypo-fractionated irradiation (4x4 Gy). The statin improved the repair of radiation-induced DNA double-strand breaks (DSBs) in all cell types and, moreover, protected lung endothelial cells from IR-induced caspase-dependent apoptosis, likely involving p53-regulated mechanisms. Under the *in vivo* situation, treatment with lovastatin or the Rac1-specific small molecule inhibitor EHT1864 attenuated the IR-induced increase in breathing frequency and reduced the percentage of *γ*H2AX and 53BP1-positive cells. This indicates that inhibition of Rac1 signaling lowers IR-induced residual DNA damage by promoting DNA repair. Moreover, lovastatin and EHT1864 protected lung tissue from IR-triggered apoptosis and mitigated the IR-stimulated increase in regenerative proliferation. Our data document beneficial anti-apoptotic and genoprotective effects of pharmacological targeting of Rho signaling following hypo-fractionated irradiation of lung cells *in vitro* and *in vivo*. Rac1-targeting drugs might be particular useful for supportive care in radiation oncology and, moreover, applicable to improve the anticancer efficacy of radiotherapy by widening the therapeutic window of thoracic radiation exposure.

Radiation therapy (RT) is a frequently used treatment modality for thoracic malignancies. However, its therapeutic efficacy is limited because of adverse effects on normal lung tissue, resulting in radiation-induced lung injury (RILI). RILI manifests as lung inflammation or fibrosis weeks to years after RT. More sophisticated RT techniques (e.g., conformal RT or intensity-modulated RT) allow better tumor control whilst sparing normal tissue.^1^ In addition, pharmacological strategies to prevent RILI are preferential because they would allow the application of higher cumulative radiation doses, thereby further improving local tumor control and, moreover, improving the quality of life of cancer patients. In view of clinical translation of preclinical data in a timely manner, off-label use of already approved drugs appears desirable.

Statins are such promising class of drugs because they are frequently used for lipid-lowering purpose and are well tolerated. All statins have in common that they interfere with the rate-limiting step in the synthesis of cholesterol by inhibiting the conversion of 3-hydroxy-3-methylglutaryl coenzyme A (HMG-CoA) by the HMG-CoA reductase. In consequence, the cellular pool of isoprene moieties (i.e., geranyl- and farnesylpyrophosphate) is depleted. In addition to cholesterol biosynthesis, isoprene residues are also required for C-terminal prenylation and membrane localization of regulatory proteins, notably Ras-homologous small GTPases (Rho-GTPases). Therefore, the pleiotropic effects of statins are believed to mainly result from the inhibition of Rho-GTPases,^2, 3^ in particular RhoA^4^ and Rac1.^5, 6^ Apart from exhibiting anticancer effects *in vitro* and *in vivo*^7, 8, 9^ statins also mitigate normal tissue damage evoked by anticancer therapeutics and irradiation. For instance, lovastatin protects cardiomyocytes from the anthracycline derivative doxorubicin.^6, 10, 11, 12^ Moreover, statins are radioprotective *in vitro*^13^ and *in vivo*.^14, 15, 16^ However, in most of these studies, the impact of statins on anticancer therapy-induced normal tissue damage was investigated in acute models, that is, after single treatment with high doses and analysis at early time points after exposure. The influence of statins on adverse late responses resulting from fractionated irradiation of the lung is unknown. Moreover, it is unclear which cell type of the lung is particularly sensitive to fractionated irradiation and, hence, might be of major relevance for the pathophysiology of RILI and, correspondingly, could be targeted for radioprotection.

In the present study we investigated (i) the effects of lovastatin on different types of confluent (non-growing) primary human lung cells following fractionated irradiation *in vitro* and (ii) the impact of lovastatin and the Rac1-specific small molecule inhibitor EHT1864 on RILI analyzed four weeks after fractionated lung irradiation *in vivo*. EHT1864 was included to figure out the specific relevance of Rac1-regulated signaling mechanisms for statin-mediated effects.

## Results

### Analysis of radioprotective effects of lovastatin in primary human lung cells

**Figure 1:**
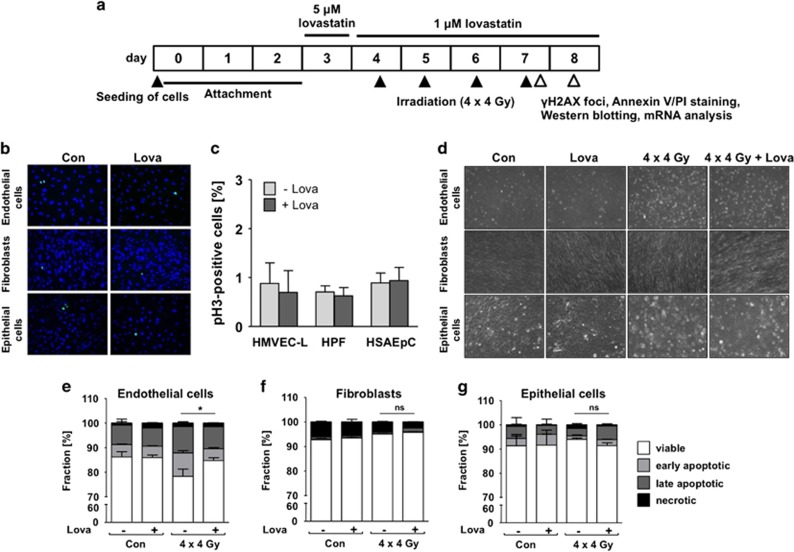
Fractionated irradiation does not induce apoptosis in confluent primary human lung epithelial cells or lung fibroblasts, but leads to apoptotic cell death in lung endothelial cells, which is prevented by lovastatin. Human microvascular endothelial cells of the lung (HMVEC-L), human pulmonary fibroblasts (HPF) and human small airway epithelial cells (HSAEpC) were seeded in high density and grown to confluency before lovastatin (Lova) (5 *μ*M) pretreatment. After incubation period of 24 h, lovastatin concentration was reduced (1 *μ*M) and irradiation was performed (4 × 4 Gy) as described in methods. Control cells were vehicle-treated and subjected to sham irradiation. (**a**) Treatment scheme. (**b**,**c**) pH3 staining (=mitotic index) after 24 h pre-incubation with lovastatin (5 *μ*M) to verify cell confluency. Shown are representative images (**b**) and quantitative data (**c**). (**d**) Analysis of cell morphology 24 h after the last irradiation. (**e**–**g**) Apoptosis was measured by Annexin V/PI staining 24 h after the last irradiation. Data show the mean±S.D. from *n*=2–3 independent experiments. Annexin V positive/PI-negative cells are considered as early apoptotic, Annexin V negative/PI-positive cells as necrotic. Two-way ANOVA with Bonferroni *post hoc* test. *P≤0.05 IR *versus* IR+Lova

**Figure 2:**
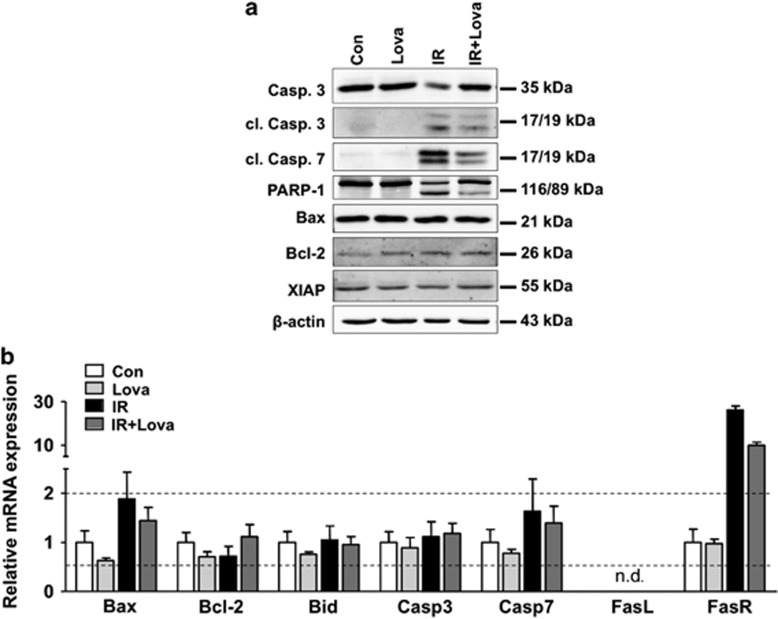
Fractionated irradiation causes activation of caspases in human lung endothelial cells, which is attenuated by lovastatin. Human microvascular endothelial cells of the lung (HMVEC-L) were seeded in high density and grown to confluency before lovastatin (Lova) (5 *μ*M) pretreatment. After incubation period of 24 h, lovastatin concentration was reduced (1 *μ*M) and irradiation was performed (4x4 Gy) as described in methods. Control cells were vehicle-treated and subjected to sham irradiation. (**a**) Protein extracts were harvested 24 h after the last irradiation and analyzed for the expression of pro-caspase 3 (Casp. 3), cleaved caspase 3 (cl. Casp. 3), cleaved caspase 7 (cl. Casp. 7), Poly(ADP-ribose)-polymerase 1 (PARP-1) as well as of Bax, Bcl-2 and XIAP. Expression of *β*-actin was used as loading control. (**b**) mRNA levels of Bax, Bcl-2, Bid, caspases 3 (Casp3) and 7 (Casp7) as well as of Fas ligand (FasL) and Fas receptor (FasR) were analyzed six hours after the last irradiation using quantitative real-time PCR. Results are shown as mean±S.D. of a representative experiment performed in triplicates. mRNA expression of ≥2 and ≤0.5 as compared with control (set to 1.0) are marked with dashed lines. n.d., not detectable

So far radioprotective effects of statins were mostly investigated under acute settings, applying single and high radiation doses. This poorly mimics the clinical relevant situation of radiotherapy. Here, we applied a hypo-fractionated irradiation protocol and analyzed delayed lung toxicity *in vitro* (Figure 1a) and *in vivo* (Figure 4b). Regarding the *in vitro* analyses, we used different types of primary human lung cells and performed the experiments at cell confluency as confirmed by microscopy (Figure 1d) and measurement of mitotic index (Figures 1b and c). Upon fractionated irradiation (4 × 4 Gy, every 24 h) of microvascular endothelial cells of the lung (HMVEC-L), pulmonary fibroblasts (HPF) or small airway epithelial cells (HSAEpC) we observed significant cytotoxicity only in HMVEC-L as shown by microscopical analysis (Figure 1d) and analysis of apoptosis by Annexin V-based method (Figures 1e–g). We assume that pulmonary fibroblasts and epithelial cells preferentially activate mechanisms of senescence following radiation.^17, 18^ Co-treatment with low doses of lovastatin completely prevented apoptosis induction in HMVEC-L (Figure 1e). Fractionated irradiation decreased the protein level of pro-caspase 3, increased the levels of cleaved caspases 3 and 7 and promoted PARP-1 cleavage (Figure 2a). Lovastatin mitigated activation of caspases and PARP cleavage (Figure 2a). Protein expression of Bax, Bcl-2 and XIAP remained unchanged in all experimental groups (Figure 2a). Fractionated irradiation-induced apoptosis of HMVEC-L was accompanied by slightly increased mRNA expression of *Bax* and substantial increase in Fas receptor (*FasR*) expression (Figure 2b), which again was partially reduced by lovastatin (Figure 2b). Overall, the data indicate that fractionated irradiation triggers caspase-dependent and lovastatin-sensitive death pathways preferentially in lung endothelial cells.

### Lovastatin protects lung cells from DNA damage induced by fractionated irradiation

**Figure 3:**
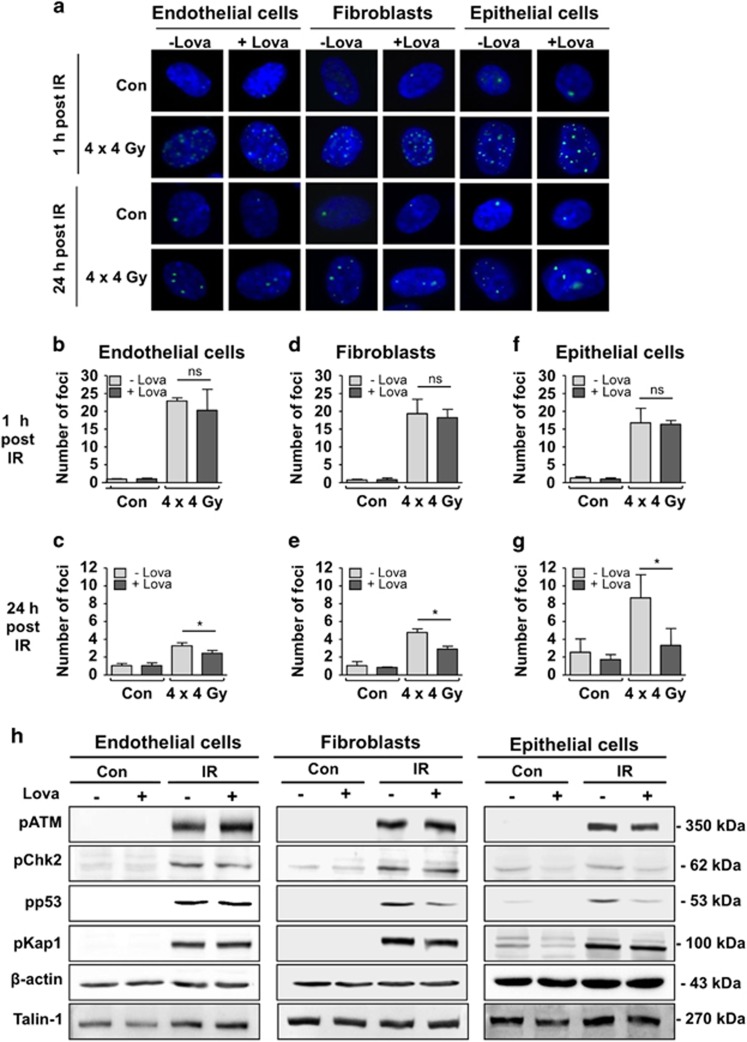
Lovastatin has no influence on initial DNA double-strand break (DSB) formation following fractionated irradiation. However, it lowers the amount of residual DNA DSBs and impacts mechanisms of the DDR in primary human lung cells. Non-growing HMVEC-L, HPF and HSAEpC were treated as described before (Figure 1a). The number of nuclear *γ*H2AX foci was analyzed by immunocytochemical staining. (**a**) Representative images. green, *γ*H2AX foci; blue, DAPI. (**b**–**g**) Number of *γ*H2AX foci detectable 1 h (**b**, **d**, **f**) and 24 h (**c**,**e**,**g**) after the last irradiation. Shown are the mean±S.D. from *n*=2–3 independent experiments. Two-way ANOVA with Bonferroni *post hoc* test. *P≤0.05 IR *versus* IR+Lova. (**h**) One hour after the last irradiation the activation status of a subset of key proteins of the DNA damage response was investigated by Western blot analysis. Shown are the protein levels of Ser1981 phosphorylated ATM (pATM), Thr68 phosphorylated checkpoint kinase-2 (pChk2), Ser15 phosphorylated protein 53 (pp53), Ser824 phosphorylated KRAB-associated protein-1 (pKap1). Expression of *β*-actin and Talin-1 were used as loading controls

DNA double-strand breaks (DSBs) are major cytotoxic lesions induced by IR and effectively trigger apoptosis or senescence.^19, 20^ Nuclear γH2AX (Ser139 phosphorylated histone H2AX) foci are commonly used surrogate markers of DSBs.^21^ All lung cell types revealed a similar initial number of γH2AX foci (Figures 3a, b, d and f), which was unaffected by the statin. Hence, we hypothesize that lung cells form identical levels of initial DSBs but differ from each other regarding complex DNA damage-triggered pro-apoptotic signaling mechanisms.^22^ After post-incubation period of 24 h, significantly lower DSB levels were found in the lovastatin co-treated groups (Figures 3c, e and g), showing that the statin accelerates the repair of DSBs. The number of residual γH2AX foci in the absence of lovastatin was 2–3-fold higher in epithelial cells as compared with endothelial cells and fibroblasts (Figures 3c, e and g), indicating that epithelial cells have the lowest DSBs repair capacity. Apparently, lovastatin favors the repair of IR-induced potentially lethal damage especially in lung endothelial cells. Lovastatin did not reduce the radiation-stimulated increase in pATM protein, a key regulator of the DNA damage response (DDR), and its substrate pKap1 (Figure 3h). Protein levels of pp53, which were also increased following IR treatment, were lower in statin-treated lung fibroblasts and epithelial cells, but were largely unaffected in endothelial cells (Figure 3h), supporting the view of cell type-specific effects of fractionated irradiation and statins on lung cells.

### Response of HUVEC and MRC-5 cells to fractionated irradiation

Including primary human umbilical vein endothelial cells (HUVEC) and human fetal lung fibroblasts (MRC-5 cells) in our study, similar results were obtained as with primary lung cells. Whereas HUVEC revealed a substantially elevated frequency of apoptotic cells following fractionated irradiation, which was reduced by lovastatin (Supplementary Figure 1A), apoptosis was not triggered in MRC-5 cells (Supplementary Figure 2A). However, both cell types showed accelerated repair of DSBs if lovastatin was present (Supplementary Figures 1B and 2B). Moreover, IR-stimulated DDR was affected by lovastatin in both cell types, as detectable on the levels of pKap1 and pp53 (Supplementary Figures 1C and 2C). The data demonstrate that the response of confluent primary human cells to hypo-fractionated irradiation is cell type-specific: a substantial apoptotic response occurs in lung microvascular endothelial and umbilical vein endothelial cells but not in lung fibroblasts or lung epithelial cells. However, independent of the cell type, lovastatin accelerates the repair of radiation-induced DSBs.

### Fractionated lung irradiation *in vivo*: effects on body weight and breathing frequency

**Figure 4:**
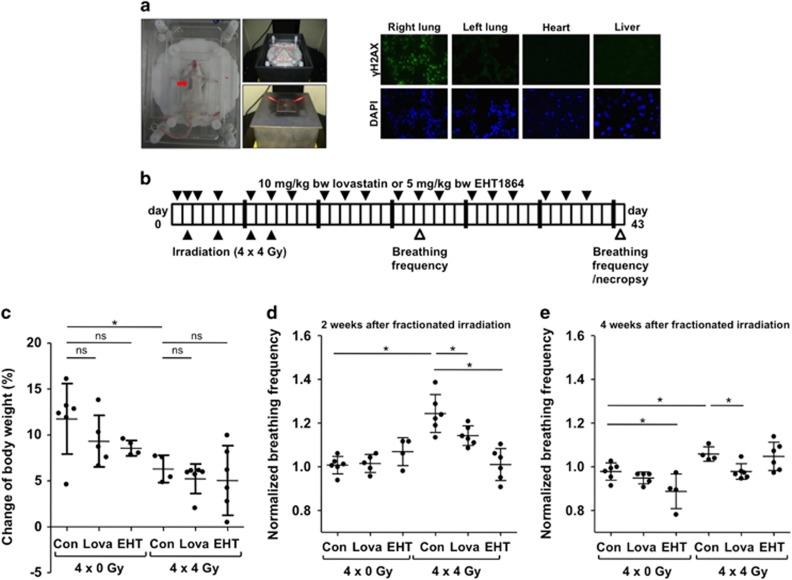
Fractionated irradiation of the lung leads to a reduced gain of body weight and an increased breathing frequency two weeks after irradiation. Lovastatin and EHT1864 treatment have no effect on body weight, but decrease IR-mediated increase in breathing frequency. (**a**) Selective irradiation of the right mouse lung was achieved using a metal-free device, which is adjustable in *x*-, *y*- and *z*-direction. 6 mm lead shielding prevents irradiation of other tissue. The red arrow marks the radiation field. One hour after single irradiation (4 Gy) of the right lung of anesthesized BALB/c mice, different tissues (lung, heart, liver) were analyzed for the presence of *γ*H2AX foci, which are indicative of DSBs. Nuclei were counterstained with DAPI. Shown are representative photographs. (**b**) Treatment scheme. As based on previous *in vivo* studies^6, 14, 66, 67^ male BALB/c mice were treated three times per week with lovastatin (10 mg/kg BW) or the Rac1-specific small molecule inhibitor EHT18641 (5 mg/kg BW) during a total period of six weeks. In the course of the first two weeks the right lung of the animals was irradiated four times with each 4 Gy (4 × 4 Gy), resulting in a cumulative dose of 16 Gy. Control animals were not irradiated (4 × 0 Gy). (**c**) Body weight was recorded three times per week. Data shown are changes in percent of body weight between the start and end of the experiment. Shown are the mean±S.D. of *n*=4–6 animals per experimental group. Two-way ANOVA with Bonferroni *post hoc* test, *P≤0.05. ns, not significant. (**d**,**e**) Breathing frequency, which is indicative of radiation pneumonitis,^24, 25, 68^ was determined two weeks (**d**) and four weeks (**e**) after the end of the fractionated irradiation. Breathing frequency of control animals was set to 1. Mean±S.D. of *n*=4–6 animals per experimental group. Two-way ANOVA with Bonferroni *post hoc* test, *P≤0.05

To address the question whether lovastatin is radioprotective *in vivo,* we aimed to selectively irradiate the right lung of mice without profuse concomitant irradiation of the left lung or other non-target organs. We established an irradiation device (Figure 4a) that is shielding other tissues (i.e., left lung, heart, liver) using CT-based 3D modeling. One hour after single irradiation (4 Gy) the frequency of *γ*H2AX-positive cells was specifically increased in the right lung but not in other adjacent tissues (Figure 4a), demonstrating the accuracy of our radiation device.

To investigate delayed radiation-induced lung injury, the right lung of male BALB/c mice was subjected to hypo-fractionated irradiation in the presence or absence of lovastatin or the Rac-specific inhibitor EHT1864^23^ (Figure 4b). Irradiation slightly slowed down the gain of body weight during the evaluation period (Figure 4c), which was not affected by lovastatin and EHT1864. The moderate radiation effect on body weight is indicative of a low general radiotoxicity. Breathing frequency, which is widely used as indication of lung function (e.g., radiation-induced pneumonitis),^24, 25, 26^ was elevated by ~20% in the irradiated group two weeks after the last irradiation (Figure 4d). While lovastatin reduced the breathing frequency only slightly, inhibition of Rac1 by EHT1864 caused a more pronounced normalization of breathing frequency (Figure 4d). Four weeks after the last irradiation, breathing frequency was only marginally elevated in the irradiated group (Figure 4e).

### Fractionated lung irradiation (4x4 Gy) does not cause sustained inflammation or oxidative stress

Lung sections stained with hematoxylin/eosin or Sirius Red showed no obvious inflammatory cell infiltrates or fibrotic lesions, respectively (Supplementary Figures 3A and B), indicating that fractionated irradiation does not cause sustained inflammatory or fibrotic processes. qRT-PCR-based mRNA expression analyses showed similar results (Supplementary Figure 4). Only IL-1*α*, MMP2 and MPO exhibited a slight mRNA upregulation in the irradiated group as compared with sham-treated controls (Supplementary Figure 4). Lovastatin and EHT1864 had no effect. A 2–3-fold increase in the number of CD68-positive cells (i.e., alveolar macrophages^27^) was observed in the irradiated group (Supplementary Figures 3C and D), which was neither influenced by lovastatin nor EHT1864 (Supplementary Figure 3D). As irradiation also triggers the generation of reactive oxygen species (ROS), we investigated oxidative stress by measuring the expression of major factors of the antioxidative defense system, which is majorly regulated by the transcription factor Nuclear Factor (erythroid-derived 2)-like 2 (Nrf2).^28^ Immunohistological staining of Nrf2 in irradiated animals did not reveal any increase in protein level (Supplementary Figure 5A). Likewise, the mRNA expression of prototypical Nrf2 target genes such as glutathione peroxidase-1 (GPX1), heme oxygenase-1 (HO-1) and glutathion-S transferase (GSTM1) remained unchanged in irradiated lung tissue (Supplementary Figure 5B). Protein and mRNA expression of manganese superoxide dismutase (MnSOD), which is radioprotective in lung,^29, 30, 31^ was also not influenced by our irradiation protocol (Supplementary Figures 5B and C). Noteworthy yet, GPX1 and HO-1 mRNA expression were upregulated in human lung endothelial cells (HMVEC-L) following fractionated irradiation *in vitro* (Supplementary Figure 5D). Lovastatin reduced the irradiation-induced upregulation of both genes in HMVEC-L. Thus, although it appears questionable that ROS is a major trigger of chronic lung toxicity following fractionated irradiation, its contribution to lung damage cannot be ruled out.

### Lovastatin and EHT1864 reduce IR-induced cell death of lung cells *in vivo*

**Figure 5:**
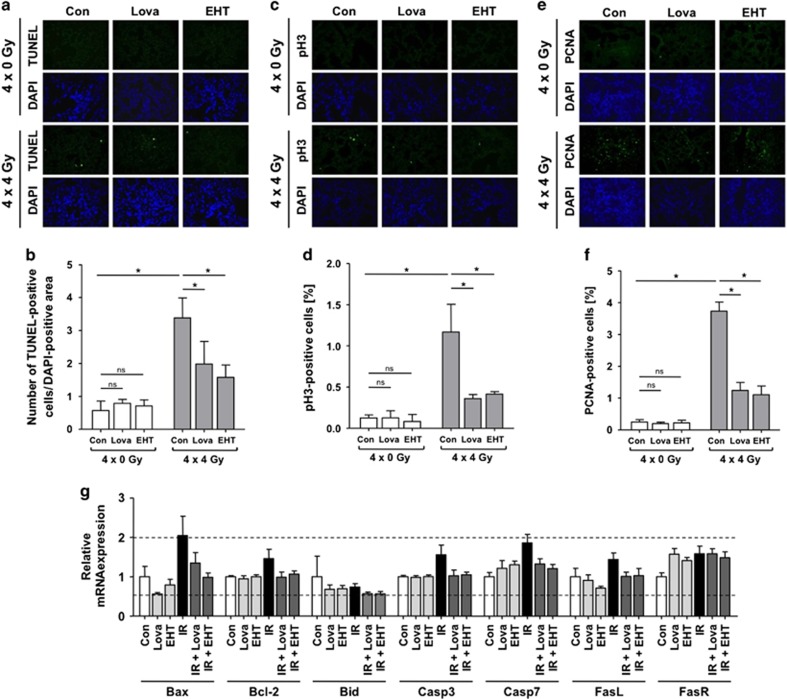
Lovastatin and EHT1864 attenuate IR-induced apoptosis and regenerative proliferation following fractionated irradiation of the lung. Male BALB/c mice were treated according to Figure 4b. Four weeks after the last irradiation the frequency of apoptotic cells (TUNEL), mitotic index (pH3) and cell proliferation (PCNA) were analyzed in the right lung. (**a**,**b**) Apoptotic frequency was determined using the TUNEL assay. (**a**) Representative image. Nuclei are stained by DAPI (blue). (**b**) Quantitative data are shown as the mean±S.D. from *n*=4–6 animals per experimental group. Two-way ANOVA with Bonferroni *post hoc* test, *P≤0.05. ns, not significant. (**c**,**d**) Mitotic index was investigated by detection of cells expressing Ser10 phosphorylated histone H3 (pH3). (**c**) Representative image. Nuclei are stained by DAPI (blue). (**d**) Quantitative data are shown as the mean±S.D. from *n*=4–6 animals per experimental group. Two-way ANOVA with Bonferroni *post hoc* test, *P≤0.05. ns, not significant. (**e**,**f**) Cell proliferation was investigated by immunohistochemistry-based staining of proliferating cell nuclear antigen (PCNA). (**e**) Representative images are shown. Nuclei are stained with DAPI (blue). (**f**) Quantitative data are shown as the mean+S.D. from *n*=3 animals per experimental group. Two-way ANOVA with Bonferroni *post hoc* test, *P≤0.05. ns, not significant. (**g**) mRNA levels of Bax, Bcl-2, Bid, caspases 3 (Casp3) and 7 (Casp7) as well as of Fas ligand (FasL) and Fas receptor (FasR) were analyzed using quantitative real-time PCR. Relative mRNA expression in non-irradiated animals was set to 1.0. mRNA expression of ≥2 and ≤0.5 as compared with control are marked with dashed lines. Shown are the mean±S.D. from pooled samples of *n*=4-6 animals per group (*N*=3)

Apoptosis of lung cells is a hallmark of RILI.^27^ Fractionated irradiation caused a 6-fold increase in the number of apoptotic (i.e., TUNEL positive) cells in the irradiated group (Figures 5a and b), which was mitigated by lovastatin and EHT1864 (Figure 5b). On protein level, no radiation-induced increase in the cleavage of caspases or PARP-1 was observed (Supplementary Figure 6B). Only the mRNA expression of *Bax* and *caspase 7* was slightly increased in irradiated animals, which was attenuated by lovastatin and EHT1864 co-treatment (Figure 5g). Fractionated lung irradiation raised the mitotic index (=frequency of phospho-histone H3 (Ser10) positive cells), which is indicative of regenerative proliferation, in the lung (Figures 5c and d). Again, both lovastatin and EHT1864 largely prevented this radiation effect. Analyzing the expression of proliferating cell nuclear antigen (PCNA), another surrogate marker of proliferation, identical results were obtained (Figures 5e and f).

### Lovastatin and EHT1864 lower IR-induced residual DNA damage in lung tissue

**Figure 6:**
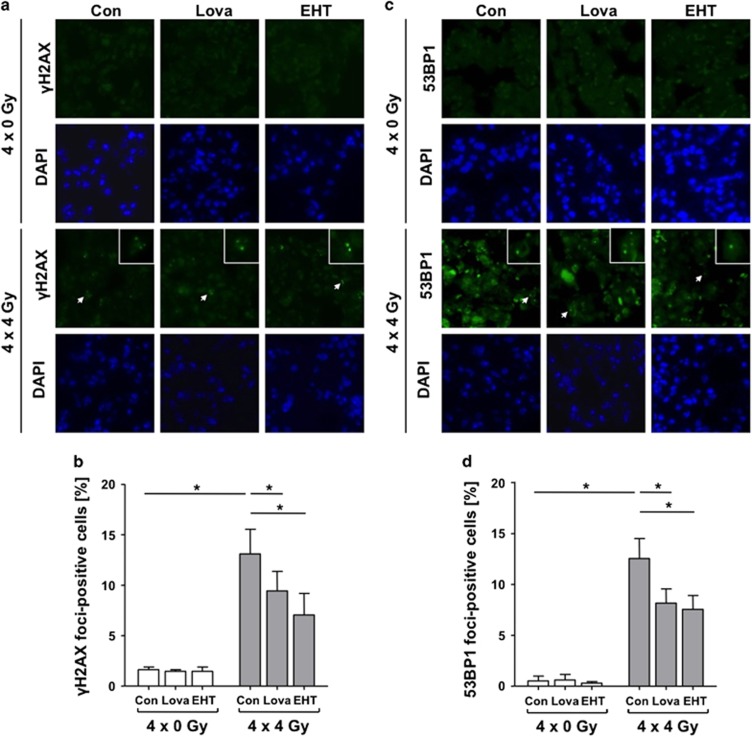
Lovastatin and EHT1864 attenuate the level of IR-induced residual DNA damage in lung tissue as reflected on the level of *γ*H2AX-positive and 53BP1-positive cells. Male BALB/c mice were treated according to Figure 4b. Four weeks after the last irradiation nuclear *γ*H2AX foci (**a**,**b**) and 53BP1 foci (**c**,**d**) were analyzed as surrogate markers of DSBs in the right lung. (**a**) Representative images. Nuclei were stained by DAPI (blue). Arrow points to a representative γH2AX-positive cell (enlarged in the image detail). (**b**) Quantification of *γ*H2AX-positive cells in tissue of the right lung. Data shown are the mean±S.D. from *n*=4-6 mice per experimental group. Two-way ANOVA with Bonferroni *post hoc* test, *P≤0.05. (**c**) Representative images are shown. Nuclei were stained by DAPI (blue). Arrow points to a representative 53BP1-positive cell (this area is enlarged in the image detail). (**d**) Quantification of 53BP1-positive cells in tissue of the right lung. Data shown are the mean±S.D. from *n*=3 mice per experimental group. Two-way ANOVA with Bonferroni *post hoc* test, *P≤0.05

IR-induced DNA double-strand breaks (DSBs) are vastly detrimental DNA lesions and effectively trigger cell death.^32^ Following fractionated lung irradiation an about 8-fold increased frequency of *γ*H2AX foci-positive cells was found (Figures 6a and b). Lovastatin reduced the IR-induced DNA damage by about 30%. This radioprotective effect was even more pronounced (~ 50%) by EHT1864. Identical results were obtained upon analysis of nuclear 53BP1 foci (Figures 6c and d), another surrogate marker of DSBs. The non-irradiated left lung showed no elevated number of *γ*H2AX foci expressing cells (Supplementary Figure 7), ruling out the possibility of delayed DNA damaging effects in non-irradiated adjacent tissue. In conclusion, lovastatin and EHT1864 lower the number of lung cells with residual DSBs resulting from fractionated irradiation.

### Influence of lovastatin and EHT1864 on IR-activated DDR *in vivo*

**Figure 7:**
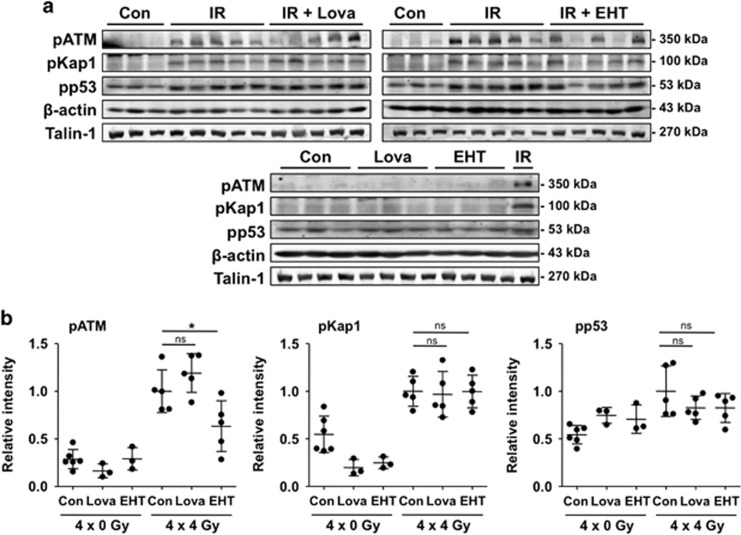
Fractionated irradiation causes a persistent activation of the DDR-related factors ATM, p53 and Kap-1 in lung tissue four weeks after the last irradiation. EHT1864 treatment significantly reduces the level of activated ATM. Male BALB/c mice were treated according to Figure 4b. Four weeks after the last irradiation the activation status of key proteins of the DNA damage response was analyzed in the right lung by western blot analysis. (**a**) Shown are the protein levels of Ser1981 phosphorylated (activated) ATM (pATM), Ser15 phosphorylated protein 53 (pp53) and Ser824 phosphorylated KRAB-associated protein-1 (pKap1). Results obtained from *n*=3–5 animals per group are presented. Expression of *β*-actin and Talin-1 were used as loading controls. (**b**) Densitometrical analysis of alterations in the protein levels of DDR factors. Relative expression in irradiated animals was set to 1.0. Data shown are obtained from *n*=3–6 animals per experimental group. Two-way ANOVA with Bonferroni *post hoc* test, *P≤0.05 (IR *versus* IR+Lova or IR *versus* IR+EHT). ns, not significant

Cells respond to the induction of DSBs with the activation of the ATM (Ataxia telangiectasia mutated) protein kinase, a key regulator of the DDR that coordinates DNA repair and apoptosis.^33, 34, 35^ The protein levels of phosphorylated ATM and its prototypical substrates p53 and the heterochromatin protein Kap-1 were elevated in total lung tissue extracts of irradiated animals as compared with non-irradiated controls (Figure 7). Protein expression of activated ATR (pATR) and checkpoint kinase 1 (pChk1), which are key players in the regulation of replicative stress responses, was not detectable under our experimental conditions (data not shown). Phosphorylation of DDR factors in the irradiated mice remained largely unaffected by lovastatin and EHT1864 (Figures 7a and b). Only the IR-induced increase in pATM was significantly reduced by EHT1864 (Figure 7b). Regarding pp53 levels, the statin and EHT caused a slight reduction (Figure 7b). Summarizing, fractionated irradiation results in a sustained activation of DDR factors in lung tissue, which is partially affected by lovastatin or EHT1864.

## Discussion

HMG-CoA reductase inhibitors (statins) have multiple cholesterol-independent effects by interfering with Rho-GTPases.^2^ Inhibition of Rho signaling by statins is radioprotective in umbilical vein endothelial cells *in vitro*^13^ and in an acute rat model of radiation enteritis,^15, 16^ with Rho-associated kinase (ROCK) being involved. Cytoprotective properties of statins on acute lung injury have been demonstrated using intratracheal instillation of the radiomimetic bleomycin,^36, 37^ single high-dose thorax irradiation^14, 38^ or total body irradiation.^39^ Here, we investigated delayed radiotoxicity resulting from fractionated irradiation employing both *in vitro* and *in vivo* model systems. We found that human lung microvascular endothelial cells are characterized by a higher frequency of radiation-induced caspase-mediated apoptosis as compared with lung fibroblasts or lung epithelial cells, indicating that especially injury of endothelial cell-related functions contributes to RILI. Since radiation-induced damage to endothelial cells is also mitigated by atorvastatin^40^ and pravastatin,^41^ we hypothesize that radiation triggered caspase-mediated death and its prevention by statins is particularly relevant for endothelial cells. By contrast, lung fibroblasts and lung epithelial cells might preferentially undergo senescence upon irradiation.^17, 18^ As fractionated irradiation-stimulated apoptosis in lung endothelial cells was accompanied by ATM-dependent activation of p53 as well as upregulation of the p53-regulated *FasR* gene, we hypothesize that p53 is involved in this process.

Lovastatin accelerated the removal of radiation-induced DSBs in all three types of lung cells, suggesting that it stimulates DSB repair. Noteworthy, lovastatin also promotes DSB repair in human keratinocytes *in vitro*.^42^ Moreover, pravastatin improves the repair of radiation-induced DSBs in fibroblasts obtained from Huntington's disease patient's^43^ and atorvastatin accelerates the repair of oxidative DNA damage in smooth muscle cells.^44^ Additionally, lovastatin prevents DSB formation following treatment with topoisomerase type II poisons.^11, 45^ Apparently, statins are genoprotective *in vitro*, which is at least partially due an improved DNA repair and/or protection from DNA damage formation. Since Rac1 is a relevant target of statins^5^ and, moreover, is present in the nucleus,^46^ we speculate that it interferes with the ATM/ATR-regulated DDR.^47^ Accordingly, pharmacological inhibition of Rac1 blocks IR-stimulated ATM/Chk2 and ATR/Chk1 activity in pancreatic carcinoma cells.^48, 49^ Interestingly, zoledronic acid, which inhibits farnesylpyrophosphate (FPP) synthesis, also promotes the repair of IR-induced DSBs in mesenchymal stem cells.^50^

To scrutinize the *in vivo* relevance of our *in vitro* data, we established a mouse model that enables a local and fractionated irradiation of the right lung and analyzed RILI at late time points (i.e., 4 weeks) after irradiation. Using this clinical designed protocol we found that inhibition of Rac1 signaling might mitigate acute radiation pneumonitis as observed two weeks after irradiation. At later time point, clear signs of persisting inflammation or sustained oxidative stress, as reflected by Nrf2-regulated activation of antioxidative defense mechanisms, were not detectable anymore. Notably, statins are known to attenuate pro-fibrotic radiation responses after single high-dose irradiation^4, 15, 16, 51, 52^ and to reduce vascular leakage and leucocyte infiltration in a model of acute RILI.^14^ Thus, our data, together with these reports, further support the hypothesis of beneficial effects of statins on RILI.

The primary cytotoxic DNA damage induced by ionizing radiation are DSBs, which stimulate DDR mechanisms regulating cell cycle checkpoints, DNA repair, cell death and senescence.^20, 33^ The interplay between mechanisms of DNA repair, DDR and apoptosis or senescence is rather complex,^22^ with p53 being a major determinant of cell fate decision, and, moreover, mechanisms of resistance are manifold.^53^ Therefore, alterations in DNA repair do not necessarily go along with proportional changes in apoptosis. This might explain the cell type-specific diverse effects of lovastatin on IR-induced apoptosis and DSB repair. We hypothesize that pharmacological interference with (early) primary stress responses resulting from radiation-induced DNA damage is more effective regarding tissue protection than abrogation of (late) secondary responses. Consequently, acceleration of DSB repair or blockage of pro-apoptotic DDR is considered as particular radioprotective. Noteworthy, lovastatin and EHT1864 significantly reduced the number of residual DSBs in lung tissue as detectable 4 weeks after the end of the fractionated irradiation. Because lovastatin does not affect the initial level of DSBs following irradiation,^13, 39^ we assume that the statin and the Rac1 inhibitor accelerate the repair of IR-induced DSBs. In consequence, a mitigated pro-apoptotic DDR and a diminished need for regenerative processes is anticipated. Indeed, we observed both a lower frequency of apoptotic cells and a reduced mitotic index in irradiated animals if Rac1 signaling was pharmacologically inhibited. Because targeting of Rho/Rac1 signaling also prevents the formation of DSBs following topoisomerase II poisoning,^11, 45^ we hypothesize that Rac1 interferes with both DNA damage induction and DNA repair, thereby eventually shutting down the primary stimulus (i.e., DNA damage) that triggers death-related DDR pathways. This hypothesis gains support by a recent report showing that epidermal Rac1 regulates DDR and repair following UV exposure, thereby protecting keratinocytes from apoptotic death.^54^

Taking into consideration the use of statins to alleviate adverse effects of radiotherapy, protection of tumor cells from DNA damage-triggered killing responses should be excluded. Fortunately, lovastatin did not attenuate IR-induced apoptosis in human breast cancer cells (MCF-7), which is a relevant cell type in the context of thoracic radiotherapy (Supplementary Figure 8). Additionally, statins augment the efficacy of various antitumor therapeutics in different tumor entities.^55, 56^ Noteworthy, pitavastatin acts as a radiosensitizer for multiple tumor cell types including lung tumor cells^57, 58, 59^ and, furthermore, radiosensitization of breast carcinoma cells^60^ and head and neck squamous cell carcinoma (HNSCC) cells^61^ is achievable by Rac1 inhibition. The molecular basis for radiosensitization of tumor cells and concomitant radioprotection of normal cells by statins and/or Rac1 inhibition remains obscure. The fact that Rho-GTPases, including Rac1, are often overexpressed in malignant tissue as compared with the corresponding normal tissue^62^ might contribute to a different response of tumor versus normal cells. In extension to data that give preference to Rho/ROCK-regulated functions for radioprotection conferred by statins,^4, 16, 51^ we provide additional evidence that inhibition of Rac1 signaling contributes to the radioprotective effects of statins on subchronic lung damage following fractionated radiotherapy. While statin-mediated inhibition of Rho/ROCK pathway impacts the late outcome of radiation injury (i.e., fibrosis),^15, 16, 51^ our data further indicate that statins also interfere with preceding radiotoxic effects (i.e., DNA damage and caspase-mediated apoptosis) by targeting Rac1 signaling. Lovastatin treatment of human endothelial cells largely reduced the level of GTP-bound active Rac1 (Supplementary Figure 9), supporting the hypothesis of Rac1 as a major target of statin-mediated effects. Collectively, the data available support the view of multiple radioprotective effects of statins on normal tissue that might be useful in the clinic to widen the therapeutic window of radiotherapy. Although animals were given higher doses of lovastatin (30 mg/kg bw/week) than used for the treatment of hypercholesterolemic patients (~5 mg/kg bw/week), even much higher statin doses (>150 mg/kg bw/week) are well tolerated in humans and do not provoke substantial adverse effects even under situation of long-term treatment.^63, 64, 65^

In conclusion, our study provides novel evidence of a radioprotective potency of lovastatin on normal primary human lung cells and rodent lung tissue under situation of hypo-fractionated irradiation, which is at least partially due to inhibition of Rac1 signaling. The data indicate that microvascular lung endothelial cells are particularly relevant for fractionated irradiation-induced lung damage and that lovastatin mediates protection from RILI. Further studies are preferential to investigate the impact of statins on chronic radiation-induced lung fibrosis. Moreover, the data encourage (i) retrospective or prospective clinical studies addressing the usefulness of statins in supportive care in radiotherapy and (ii) the development and preclinical analysis of novel Rac1-specific inhibitory drugs for radioprotection.

## Materials and methods

### Cell culture conditions and treatment of human cells

HMVEC-L were obtained from Lonza (Basel, Switzerland) and cultured in EGM-2MV medium. HPF and HSAEpC were purchased from PromoCell (Heidelberg, Germany) and cultured in Fibroblast Growth Medium 2 and Small Airway Epithelial Cell Growth Medium obtained from the provider, respectively. The cells were directly obtained from the aforementioned providers at passage P2 and were cultured in our laboratory for less than 6 months and <15 population doublings. HMVEC-L were authenticated by the provider by monitoring the expression of von Willebrand Factor VIII and PECAM as well as the uptake of acetylated low density lipoprotein. Also HSAEpC and HPF authentication was performed by the provider. Here, cell morphology and FACS-based analysis of cytokeratin (HSAEpC) and CD90 (HPF) expression were monitored. Human umbilical vein endothelial cells (HUVEC) and human breast cancer cells (MCF-7) originate from PromoCell and and the German Tissue Culture Collection (DSMZ, Braunschweig, Germany), respectively, and were cultured in Endothelial Cell Growth Medium 2 and Dulbecco's modified Eagle medium (Sigma, Steinheim, Germany), respectively. Human lung fibroblasts (MRC-5) cells were purchased from CLS Cell Lines Service (Heidelberg, Germany) and cultivated in Dulbecco's modified Eagle medium:Ham’s F12 (1:1) (Biochrom, Berlin, Germany). Cells were kept at 37 °C in a humidified atmosphere containing 5% CO_2_. They were seeded in high density and grown for 72 h to ensure confluency before pretreatment with 5 *μ*M lovastatin (Sigma). The experiments were done at cell confluency because this reflects the *in vivo* situation. 24 h later medium was changed and lovastatin concentration was reduced to 1 *μ*M and cells were irradiated on four days in a row with 4 Gy (^137^Cs source (Gammacell 3000, Nordion, Ottawa, Canada)). This experimental setup was chosen to analyze the ability of lovastatin to favor the repair of potentially lethal damage (PLD) induced by fractionated irradiation.

### Analysis of nuclear γH2AX foci formation and determination of mitotic index

The formation of Ser139 phosphorylated histone 2AX nuclear foci (γH2AX foci) was measured as surrogate marker of ionizing radiation (IR)-induced DNA double-strand breaks (DSBs) by immunofluorescence. Ser10 phosphorylation of histone H3 is a commonly used marker of mitotic index. Cells were fixed with 4% formaldehyde followed by overnight incubation with methanol (-20 °C). After blockage (5% BSA in 0.3% Triton X-100/PBS), cells were incubated with anti-Ser139 phosphorylated histone 2AX (γH2AX) antibody (1:500, 16 h, 4 °C) (Millipore, Billerica, MA, USA) or anti-Ser10 phosphorylated histone 3 antibody (pH3) (1:200, 16 h, 4 °C) (Thermo Fisher Scientific, Waltham, MA, USA). After washing, Alexa Fluor 488 goat anti-mouse IgG antibody (Rockland, Limerick, PA, USA) was added (90 min, RT). Cell nuclei were counterstained with DAPI-containing Vectashield (Vector Laboratories, Burlingame, CA, USA) and γH2AX foci or pH3 positive cells were detected using Olympus BX43 microscope (Olympus, Hamburg, Germany).

### Measurement of apoptotic cell death by Annexin V/PI staining

Apoptosis was analyzed by flow cytometer-based Annexin V/PI method. To this end, cells were resuspended in Annexin binding buffer (10 mM HEPES, 140 mM NaCl, 2.5 mM calcium chloride) and 10^5^ cells were incubated with 5 *μ*l Fluorescein-conjugated Annexin V (Life Technologies, Carlsbad, CA, USA) (15 min, on ice). 10 *μ*l propidium iodide solution (50 *μ*g/ml) and 200 *μ*l Annexin binding buffer were added before samples were analyzed by BD Accuri™ C6 flow cytometer (BD, Franklin Lakes, NJ, USA) (530/30 nm and 585/40 nm filters). Cell morphology was monitored via light microscopy (Axiovert 40 CFL ((Zeiss, Jena, Germany)).

### Animal experiments

Male BALB/c mice (12–16 weeks old, 20–30 g) were kept in the local animal housing facility of the University Hospital Düsseldorf (Germany). All animal experiments were approved by the local authorities (i.e., State Agency for Nature, Environment and Consumer protection, North Rhine-Westfalia, Germany; Reference number: 84-02.04.2013.A062) and performed according to the relevant guidelines and regulations. A total of six animals were randomized to each group. In line with previous studies^6, 14, 66, 67^ animals were treated three times per week with lovastatin (10 mg/kg bw, p.o) (Betapharm Arzneimittel GmbH, Augsburg, Germany) or the Rac1 small molecule inhibitor EHT1864 (5 mg/kg bw, i.p.) (Tocris Bioscience, Bristol, UK) starting two days before the first irradiation dose and continuing till the end of the experiment (Figure 4b). Vehicle-treated animals were used as control. Body weights were recorded three times per week. Fractionated local irradiation of the right lung was performed four times with each 4 Gy (cumulative dose: 16 Gy; biological effective dose (BED): 37.3 Gy) within two weeks. Before irradiation mice were anesthetized with ketamine (100 mg/kg bw) and xylazin (5 mg/kg bw). As irradiation device a Gulmay RS 225 (15 mA, 200 kV) was used. For the selective irradiation of the right lung, mice were adjusted into an irradiation device that allows selective irradiation of the right lung (Figure 4a) (radiation field: 1.26 cm^2^). Control mice were subjected to sham irradiation.

### Analysis of breathing frequency

Breathing frequency was monitored two and four weeks after the last irradiation. To this end, mice were anesthetized as described above and breathing frequency was determined 15 min later by two independent investigators (recording for one minute and calculation of mean values).

### Histological and immunohistochemical analyses

Formaldehyde-fixed paraffin-embedded lung tissue samples were cut into 4 *μ*m sections. Paraffin removal and tissue rehydration was performed according to standard procedures. To evaluate inflammation and fibrosis, sections were stained with hematoxylin/eosin (HE) (Sigma–Aldrich, Steinheim, Germany) and Sirius Red (Waldeck, Münster, Germany), respectively. For Nrf2 staining sections were incubated in 3% H_2_O_2_ for 20 min, boiled in Target Retrieval Solution (DAKO, Hamburg, Germany) for 30 min and blocked in TNB buffer (PerkinElmer, Waltham, MA, USA) for 30 min. Afterwards, samples were incubated with 0.001% avidin and 0.001% biotin (15 min each) before rabbit anti-Nrf2 antibody (1:1000; Santa Cruz, CA, USA) was added (4 °C overnight). After washing, sections were incubated with biotinylated donkey anti-rabbit IgG (Santa Cruz, CA, USA) (1:200, 45 min at RT). Afterwards TSA Biotin Kit (PerkinElmer) was used according to the manufacturer’s instructions and sections were incubated with ABC reagent (Vectastain Elite ABC HRP Kit, Vector Laboratories, Burlingame, CA, USA) and stained with 3,3’-diaminobenzidine (DAB Peroxidase Substrate Kit (Vector Laboratories)) for 90 s. Sections were counterstained with hematoxylin, mounted in Entellan and evaluated using Olympus BX43 microscope. For immunofluorescent stainings epitopes were demasked by incubation with Target Retrieval Solution (DAKO, Hamburg, Germany) in a steam boiler (1 h) before blocking in Protein Block (DAKO, Hamburg, Germany) (1 h). Incubation with primary antibody was performed overnight (4 °C, wet chamber). The following antibodies were used: rabbit anti-Ser139 phosphorylated histone 2AX (1:400 Abcam, Cambridge, MA, USA) and anti-53BP1 (1:400, Cell Signaling, Beverly, MA, USA) to detect DNA damage; rabbit anti-Ser10 phosphorylated histone 3 (1:250, Thermo Fisher Scientific) to monitor mitotic index; mouse anti-proliferating cell nuclear antigen (PCNA) (Millipore) to analyze proliferation, rabbit anti-CD68 (1:100, Abcam) to detect macrophages. After washing (PBS/0.1% Tween 20 (3 × 5 min)) Alexa Fluor 488-coupled goat anti-rabbit secondary antibody (Invitrogen, Darmstadt, Germany) was used. Sections were mounted in DAPI-containing Vectashield and evaluated with Olympus BX43 microscope.

### Analysis of apoptosis by TUNEL assay

Apoptotic cells were detected by the *In Situ* Cell Death Detection Kit Fluorescein (Roche, Mannheim, Germany) according to the manufacturer’s instructions. Protein digestion was performed with 20 *μ*g/ml Proteinase K (Qiagen, Hilden, Germany). As positive control treatment with 150 U/ml DNase (Qiagen) was performed. Nuclei were counterstained with DAPI-containing Vectashield and analyzed with Olympus BX43 microscope.

### Total RNA purification, cDNA synthesis and qRT-PCR analysis

Total RNA from 10–20 mg lung tissue or 1–5 × 10^6^ cells was isolated using the RNeasy Mini Kit (Qiagen). RNA yield and purity were determined with NanoVue Plus Spectrophotometer (GE Healthcare, Freiburg, Germany). RNA was isolated from *n*=4–6 animals per experimental group and pooled in equal amounts for cDNA synthesis using High-Capacity cDNA Reverse Transcription Kit (Applied Biosystems, Darmstadt, Germany). For each cDNA synthesis reaction 1000–2000 ng of total RNA was used. Quantitative real-time PCR analysis (triplicate determinations) was accomplished with 20 ng cDNA using CFX96 Touch Real-Time PCR Detection System (Bio-Rad Laboratories, Hercules, CA, USA). A semi-customized PCR-array (Sigma–Aldrich) containing 29 selected genes (Supplementary Table 1) involved in inflammation and fibrosis was used for quantitative mRNA analyses. Moreover, primers for selected oxidative stress and apoptosis-related genes were synthesized by Eurofins (Ebersberg, Germany). mRNA levels of target genes were normalized to that of the housekeeping genes glyceraldehyde 3-phosphate dehydrogenase (GAPDH), beta actin (*β*-actin) and ribosomal protein L32 (RPL32). 45 amplification cycles were performed (each cycle: 95 °C – 15 s; 55 °C – 15 s; 72 °C – 17 s). Following each run a melt curve analysis was included to ensure product specificity. PCR products with threshold cycles of ≥36 were omitted. Gene expression of control animals was set to 1. Data analysis was performed with the CFX Manager Software (Bio-Rad Laboratories). Alterations in mRNA expression of ≥2 and ≤0.5 as compared with untreated control animals (set to 1.0) are considered as particularly relevant and are marked with dashed lines.

### Preparation of protein extracts and western blot analysis

To prepare total protein extracts 10–20 mg lung tissue was homogenized (lysis buffer: 50 mM Tris HCl, 150 mM NaCl, 2 mM EDTA, 1% NP 40, 0.1% sodium dodecyl sulfate, 1% sodium desoxycholate, 1 mM sodium orthovanadate, 1 mM phenylmethylsulfonyl fluoride, 50 mM sodium fluoride and 1 x Protease inhibitor cocktail (Cell Signaling)) with Tissue Lyzer II (Qiagen) and sonicated on ice before centrifugation (10 min at 10.000 × *g*, 4 °C). Protein concentration of the supernatant was determined with the DC Protein Assay (Bio-Rad Laboratories). Protein samples were diluted with Roti-Load 1 (Roth, Karlsruhe, Germany) and heated (95 °C, 5 min). 30–100 *μ*g of total protein was separated by SDS-PAGE (6–15% gels) and transferred onto nitrocellulose membranes (GE Healthcare, Freiburg, Germany) using Mini-PROTEAN electrophoresis chamber (Bio-Rad Laboratories). After blocking of membranes (5% dry milk in TBS/0.1% Tween 20, 2 h), incubation with primary antibody (1:1000) was performed (overnight, 4 °C). After incubation with HRP-conjugated secondary antibodies (1:2000, Rockland Immunochemicals Inc., Limerick, Pam USA) (2 h, RT), bound antibodies were visualized by chemiluminescence using the Fusion FX7 imaging system (Vilber Lourmat, Eberhardzell, Germany) or ChemiDoc Imaging System (Bio-Rad Laboratories). The following primary antibodies were used: anti-Ser15 phosphorylated protein 53 (pp53), anti-Ser345 phosphorylated checkpoint kinase 1 (pChk1) anti-caspase 3, anti-activated caspase 3 and 7, Talin-1 (Cell Signaling), anti-Ser824 phosphorylated KRAB-associated protein-1 (pKap1) (Bethyl Laboratories Inc., Montgomery, TX, USA), anti-Ser1981 phosphorylated Ataxia telangiectasia mutated (pATM), anti-Thr68 phosphorylated checkpoint kinase-2 (pChk2), (Abcam), anti-Bax, anti-Bcl-2, anti-XIAP, anti-*β*-actin and anti-Poly(ADP-ribose)-polymerase 1 (PARP-1) antibodies (Santa Cruz).

### Active Rac1 pull-down assay

The level of active Rac1 was analyzed by Rac1 pull-down assay, which measures the binding of GTP-bound (active) Rac1 to its effector protein p21-associated protein kinase 1 (PAK1). To this end, GST-fused PAK1-GTPase-binding domain, which was isolated after recombinant expression in *Escherichia coli*, was used. GSH sepharose beads (GE Healthcare, Little Chalfont, UK) were washed three times with 1 ml Fish buffer (50 mM Tris/HCl pH 7.4, 100 mM NaCl, 2 mM MgCl_2_, 1% Igepal Ca-630, 10% glycerol, 20 mM beta-glycerophosphate, 1 mM sodium orthovanadate Na_3_VO_4_, 1 tablet complete, EDTA-free Protease Inhibitor Cocktail (Roche, Basel, Switzerland)) and equal amounts of bacterial lysates containing GST (negative control) or GST-fused PAK1-GTPase-binding domain were added to the beads and incubated for 1 h at 4 °C, followed by another three times washing with Fish buffer. HUVEC total cell lysate was obtained from two confluent 10 cm dishes, added to the GST or GST-PAK1-bound sepharose beads and incubated for 30 min at 4 °C. The samples were washed three times with Fish buffer and Roti-Load 1 (Roth, Karlsruhe, Germany) was added to the samples before heat denaturation for 10 min at 95 °C was performed. Samples were analyzed by SDS-PAGE and immunoblotting as described above. Mouse anti-Rac1 antibody (1:2500) from Millipore was used for the detection of Rac1.

### Statistical analysis

Results are expressed as mean±S.D. For statistical analysis, experimental groups were compared by two-way analysis of variance (ANOVA) with Bonferroni *post hoc* test using GraphPad Prism 5 (GraphPad Software Inc., La Jolla, CA, USA). A *P*-value of *P*≤0.05 was defined as level of significance.
