# Supplementary material for: Rho inhibition by lovastatin affects apoptosis and DSB repair of primary human lung cells in vitro and lung tissue in vivo following fractionated irradiation
Source: Cell Death Dis. 2017 Aug 10;8(8):e2978–. doi: 10.1038/cddis.2017.372 (PMC5596560; doi:10.1038/cddis.2017.372)
Supplement: Supplementary Figures and Table [file cddis2017372x1.doc]

**Supplementary Information**

**Rho inhibition by lovastatin affects apoptosis and DSB repair of primary human lung cells *in vitro* and lung tissue *in vivo* following fractionated irradiation**

Verena Ziegler^1^, Christian Henninger^1^, **Ioannis Simiantonakis**^2^**,** Marcel Buchholzer^3^, **Mohammad** Reza Ahmadian^3^, Wilfried Budach^2^, Gerhard Fritz^1*^

^1^Institute of Toxicology, Medical Faculty, Heinrich Heine University, Duesseldorf, Moorenstrasse 5, 40225 Duesseldorf, Germany

^2^Department of Radiotherapy and Radiation Oncology, University Hospital Duesseldorf, Moorenstraße 5 , 40225 Duesseldorf, Germany

^3^Institute of Biochemistry and Molecular Biology II, Medical Faculty, Heinrich Heine University, Duesseldorf, Germany

^*^Corresponding author:

Gerhard Fritz, Ph.D.

Institute of Toxicology

Heinrich Heine University Düsseldorf

Universitätsstrasse 1

40225 Düsseldorf

Phone: +49-221-81-13022

Fax: +49-221-81-13013

E-mail: fritz@uni-duesseldorf.de

**Supplementary figure S1: Lovastatin prevents IR-induced apoptosis in human endothelial cells (HUVEC) and interferes with DNA damage response and repair as reflected on the levels of altered activation status of DDR proteins and reduced number of DNA DSBs, respectively.**

Human Umbilical Vein Endothelial Cells (HUVEC) were seeded in a high density and were grown to confluency before lovastatin treatment and fractionated irradiation started as described in Figure 1A.

A: Apoptosis was measured by Annexin V/PI staining 24 h after irradiation. Data show the mean ± SD from n=2-3 independent experiments. Annexin V positive/ PI negative cells are considered as early apoptotic, Annexin V negative/PI positive cells as necrotic. Two-way ANOVA with Bonferroni post-hoc test. * p ≤ 0.05 IR vs. IR+Lova.

B: Number of γH2AX foci detectable 1 h and 24 h after the last irradiation. Shown are the mean ± SD from n = 2-3 independent experiments. Two-way ANOVA with Bonferroni post hoc test. * p ≤ 0.05 IR vs. IR+Lova. n.d., not determined.

C: One hour after the last irradiation the activation status of a subset of key proteins of the DNA damage response was investigated by Western blot analysis. Shown are the protein levels of Ser1981 phosphorylated ATM (pATM), Thr68 phosphorylated checkpoint kinase‑2 (pChk2), Ser15 phosphorylated protein 53 (pp53) and Ser824 phosphorylated KRAB-associated protein‑1 (pKap1). Expression of Talin‑1 was used as loading control.

**Supplementary figure S2:**  **Fractionated irradiation induces DNA DSBs and activates DDR mechanisms in human fetal lung fibroblasts (MRC-5) without concomitant induction of cell death. Lovastatin reduces the number of residual DNA DSBs and alters the activation status of DDR key proteins.**

Human MRC-5 lung fibroblasts (MRC-5) were seeded in a high density and were grown to confluency before lovastatin treatment and fractionated irradiation started as described in Figure 1A.

A: Apoptosis was measured by Annexin V/PI staining 24 h after irradiation. Data show the mean ± SD from n=2-3 independent experiments. Annexin V positive/ PI negative cells are considered as early apoptotic, Annexin V negative/PI positive cells as necrotic. Two-way ANOVA with Bonferroni post-hoc test. * p ≤ 0.05 IR vs. IR+Lova.

B: Number of γH2AX foci detectable 1 h and 24 h after the last irradiation. Shown are the mean ± SD from n = 2-3 independent experiments. Two-way ANOVA with Bonferroni post hoc test. * p ≤ 0.05 IR vs. IR+Lova. n.d., not determined.

C: One hour after the last irradiation, the activation status of a subset of key proteins of the DNA damage response was investigated by Western blot analysis. Shown are the protein levels of Ser1981 phosphorylated ATM (pATM), Thr68 phosphorylated checkpoint kinase‑2 (pChk2), Ser15 phosphorylated protein 53 (pp53) and Ser824 phosphorylated KRAB-associated protein‑1 (pKap1). Expression of Talin‑1 was used as loading control.

**Supplementary figure S3: Fractionated irradiation does not lead to acute lung inflammation or substantial lung fibrosis as analyzed four weeks after irradiation.**

Male BALB/c mice were treated according to Fig. 4B.

A, B: Hematoxylin & Eosin (HE) staining (A) and Sirius Red staining (B) of right lung tissue four weeks after the end of fractionated irradiation with 4 x 4 Gy. A: 20 and 100 x objective; B: 10 and 40 x objective. Data shown are representative images from n = 4-6 animals per group.

C: Representative images of CD68-positive cells in non-irradiated (Con) and irradiated (IR) lung tissue four weeks after fractionated irradiation with 4 x 4 Gy.

D: Quantification of CD68-positive cells from n = 4 animals per experimental group. Results are expressed as mean ± SD.

**Supplementary figure S4: Fractionated irradiation does not affect RNA expression of inflammation- and fibrosis-related genes at late times after fractionated irradiation.**

Male Balb/c mice were treated according to Fig. 4B. Four weeks after the last irradiation, mRNA levels of pooled RNA samples from n=4-6 animals per group were analyzed using a semi-customized, quantitative Real-Time PCR-array as described in methods.

A: Scatter plot illustrating differences in gene expression observed in control vs. irradiated group.

B: Alterations in gene expression of representative genes coding for factors involved in inflammation and fibrosis. mRNA expression of ≥2 and ≤0.5 as compared to control are marked with dashed lines. Shown are the mean ± SD from n = 4-6 animals per group, N=3.

**Supplementary figure S5: Fractionated irradiation does not alter the mRNA expression of oxidative stress-associated genes in lung tissue but increases mRNA expression of GPX and HO-1 in human lung endothelial cells.**

Male Balb/c mice were treated according to Fig. 4A and lung tissue was harvested four weeks after the last irradiation (A-C).

A: Nrf2 staining of lung tissue of irradiated and sham-irradiated animals four weeks following fractionated irradiation. Nrf2-positive cells are stained in brown color. Representative images are shown.

B: mRNA levels of glutathione peroxidase-1 (GPX), heme oxygenase-1 (HO-1), manganase superoxide dismutase (MnSOD) as well as glutathion-S transferase (GSTM1) were analyzed using quantitative Real-Time PCR. Relative mRNA expression in non-irradiated animals was set to 1.0. Shown are the mean ± SD from pooled samples of n = 4-6 animals per group (N=3).

C: Shown are the protein levels of manganase superoxide dismutase (MnSOD) in irradiated and sham-irradiated animals four weeks following fractionated irradiation. Results obtained from n = 3 animals per group are presented. Expression of β-actin was used as loading control. mRNA expression of ≥2 and ≤0.5 as compared to control (Con; 1.0) is marked with dashed lines.

D: Human Microvascular Endothelial Cells of the Lung (HMVEC-L) were seeded in high density and grown to confluency before lovastatin treatment and fractionated irradiation started as described in Figure 1A. mRNA levels of glutathione peroxidase-1 (GPX), heme oxygenase-1 (HO-1), manganase superoxide dismutase (MnSOD) as well as glutathion-S transferase (GSTM1) were analyzed six hours after the last irradiation using quantitative Real-Time PCR. Results are shown as mean ± SD from one representative experiment performed in triplicate. Alterations in mRNA expression of ≥2 and ≤0.5 are marked with dashed lines.

**Supplementary figure S6: Fractionated irradiation of mouse lung does not trigger substantial activation of caspases.**

Male Balb/c mice were treated according to Fig. 4A and lung tissue was harvested four weeks after the last irradiation.

Shown are the protein levels of pro-caspase 3 (Casp. 3), cleaved caspase 3 (cl. Casp. 3), cleaved caspase 7 (cl. Casp. 7), Poly(ADP-ribose)-polymerase 1 (PARP-1) as well as Bax, Bcl-2 and XIAP in irradiated and sham-irradiated animals four weeks following fractionated irradiation. Data presented were obtained from n = 3 animals per group. Expression of β-actin was used as loading control.

**
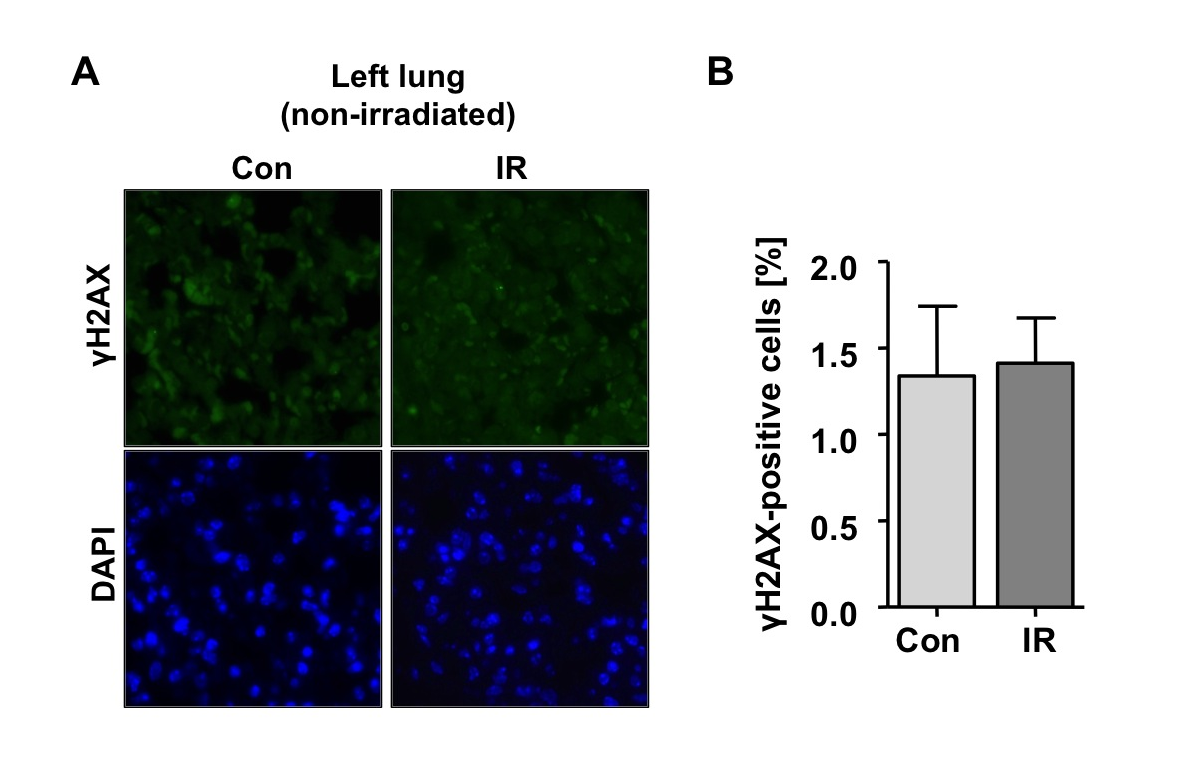
**

**Supplementary figure S7: Irradiation of the right lung does not lead to increased γH2AX levels in the left lung.**

The right lung of male Balb/c mice was irradiated four times with 4 Gy (IR) during a period of two weeks. Control animals (Con) were sham-irradiated. Four weeks after the last irradiation the number of γH2AX positive cells was analyzed in the left lung tissue.

A: Representative picture. Nuclei are stained with DAPI (blue).

B: Quantitative data shown are the mean ± SD from n = 3 animals per group.

**Supplementary figure S8: Lovastatin does not protect human breast cancer cells (MCF-7) from fractionated irradiation-induced apoptotic cell death.**

MCF-7 cells were pretreated with lovastatin (5 μM). After incubation period of 24 h, lovastatin concentration was reduced (1 μM) and irradiation was performed (4 x 4 Gy). Control cells were vehicle-treated and subjected to sham-irradiation. Apoptosis was measured by FACS-based Annexin V/PI staining 24 h after the end of the irradiation. Data show the mean ± SD from n=3 independent experiments.

**Supplementary figure S9: Lovastatin treatment reduces the protein level of active, GTP-bound Rac1 in human endothelial cells**.

Human Umbilical Vein Endothelial Cells (HUVEC) were seeded in a high density and were grown to confluency before cells were treated with 5 μM lovastatin (Lova) for 24 h. Afterwards, cells were harvested and subjected to Rac1 pull-down assay using GST-PAK1 as described in methods. GST was used as negative control, recombinant Rac1-GDP and non-hydrolyzable Rac1-GTP were used as positive controls.

Supplementary table 1: List of genes that were analyzed by qRT-PCR analysis.

| ACTA2 | actin, alpha 2, smooth muscle |
| --- | --- |
| ACTB | actin, beta |
| ALOX5 | arachidonate 5-lipoxygenase |
| CCL2 | chemokine (C-C motif) ligand 2 |
| CD68 | CD68 antigen |
| CDH1 | cadherin 1, type 1, E-cadherin |
| COL1A1 | collagen, type I, alpha 1 |
| COL1A2 | collagen, type I, alpha 2 |
| COL3A1 | collagen, type III, alpha 1 |
| COX-2 | prostaglandin-endoperoxide synthase 2 |
| CTGF | connective tissue growth factor |
| CXCL2 | chemokine (C-X-C motif) ligand 2 |
| GAPDH | glyceraldehyde-3-phosphate dehydrogenase |
| IFNG | interferon, gamma |
| IL-1α | interleukin 1, alpha |
| IL-1β | interleukin 1 beta |
| IL-6 | interleukin 6 |
| IL-10 | interleukin 10 |
| IL-13 | interleukin 13 |
| MMP9 | matrix metallopeptidase 9 |
| MMP2 | matrix metallopeptidase 2 |
| MPO | myeloperoxidase |
| NLRP3 | NLR family, pyrin domain containing 3 |
| PTPRC | protein tyrosine phosphatase, receptor type, C (CD45) |
| SFTPC | surfactant associated protein C |
| SMAD2 | SMAD family member 2 |
| SNAI1 | snail family zinc finger 1 |
| RPL32 | ribosomal protein L32 |
| TGF-ß1 | TGFB1 transforming growth factor, beta 1 |
| TIMP1 | TIMP metallopeptidase inhibitor 1 |
| TNF | tumor necrosis factor |
| VIM | Vimentin |
